# Risk of death in crashes on Ontario’s highways

**DOI:** 10.1186/1471-2458-12-1125

**Published:** 2012-12-28

**Authors:** Damian Rzeznikiewiz, Hala Tamim, Alison K Macpherson

**Affiliations:** 1 School of Kinesiology and Health Science, York University, 4700 Keele Street, Toronto, M3J 1P3, Canada

## Abstract

**Background:**

Motor vehicle collisions (MVCs) that result in one or more fatalities on the 400-series Highways represent a serious public health problem in Ontario, and were estimated to have cost $11 billion in 2004. To date, no studies have examined risk factors for fatal MVCs on Ontario’s 400 series highways.

The investigate how demographic and environmental risk factors are associated with fatal MVCs on Ontario’s 400-Series Highways.

**Methods:**

Data were provided from the Ontario Ministry of Transport database, and included driver demographics, vehicle information, environmental descriptors, structural descriptors, as well as collision information (date and time), and severity of the collision. Multivariate analysis was used to identify factors significantly associated with the odds of dying in a collision.

**Results:**

There were 53,526 vehicles involved in collisions from 2001 to 2006 included in our analysis. Results from the multivariate analysis suggest that collisions with older age and male drivers were associated with an increased risk of involving a fatality. Highway 405 and an undivided 2-way design proved to be the most fatal structural configurations. Collisions in the summer, Fridays, between 12 am-4 am, and in drifting snow conditions during the wintertime were also shown to have a significantly increased risk of fatality.

**Conclusion:**

Our results suggest that interventions to reduce deaths as a result of MVCs should focus on both driver-related and road-related modifications.

## Background

Unintentional injuries, many of which are attributable to motor vehicle collisions, are the 6th leading cause of death for Canadians [1]. With close to nine million drivers on Ontario’s roads, the costs arising from motor vehicle collisions (MVCs) on the 400-series highways in Ontario which result in one or more fatalities are immense when all aspects are taken into account. A report released by the Ministry of Transportation of Ontario (MTO) in 2007, calculated the social costs of MVCs to be $18 billion in the year 2004 [2]. Additionally, fatalities were reported to have cost $11 billion (64% of the total social costs) even though fatal collisions represented less than 1% of the 231,548 Highway Traffic Act reportable collisions in 2004. The average social cost of a fatal collision in 2004 was $15.7 million.

Conversely, even though the costs of MVCs are astonishing, it is important to highlight the advances the province has made in road safety, which include a decline in fatality rates over the past three decades. Since 1980, the number of licensed drivers has increased by almost 80% but the number of fatalities has decreased by 49% [3]. However, in the six-year period spanning from 2001 to 2006, there were 815 fatal MVCs on Ontario’s 400-Series Highways that in turn cost the province $12.8 billion, or almost $2.6 billion per year.

As a result of these astounding costs, the province has invested and continues to invest billions of dollars every year in engineering and restructuring plans in order to improve our highways and subsequently decrease the number of MVCs.

There has been previous research investigating driver characteristics and environmental risk factors associated with fatal motor vehicle collisions in Ontario. For example, studies suggest that the older driver population is at an elevated risk for being involved in fatal MVCs [4,5]. Further, younger drivers are at an elevated risk as well due to their risk-taking behavior which may be expressed by going at higher speeds or by using alcohol and/or drugs when driving [6-8].

In addition to age, sex has been studied as a possible risk-factor for the involvement in fatal MVCs. Zhang found no difference between male and female fatality rates in MVCs [9]. Interestingly, Singleton et al. found that females were 60% more likely than males to be seriously injured in MVCs, and Bedard et al. explained that females in the United States were 54% more likely to be involved in a fatal accident than their male counterparts [10,11]. Moreover, Lemieux found that when looking at MVCs in the Hamilton region of Ontario, weather did not play a significant role in the collisions that resulted in one or more fatalities [12].

### Objective

To date, there have been no studies conducted which examine Ontario’s 400-Series highways and the risk of dying in a crash. As a result, the objective of this study is to investigate how driver demographics and environmental risk factors are associated with fatal motor vehicle collisions on Ontario’s 400-Series Highways.

## Methods

### Data

Road Traffic Accident Data from the Ministry of Transportation of Ontario (MTO) included all Highway Traffic Act (HTA) reportable accidents on Ontario 400-Series highways between January 1st, 2001, and December 31st, 2006. The data, compiled by MTO, are composed of information extracted from police reports of MVCs that take place on the province’s 400-Series highway system. These controlled-access freeways are similar to the interstate network in the United States and are located in the southern and central regions of Ontario, Canada. All of the highways are at least four lanes, have posted speed limits of between 80 and 100 kilometres an hour, and generally restrict access to pedestrians and non-motorized vehicles. No data that could identify anyone involved in the collisions were requested; therefore approval from an Ethics Committee was not necessary. We used Highway 401 as the reference group because it included the largest number of collisions.

A total of 53,526 vehicles involved in collisions from 2001 to 2006 were included in our analysis. Two-wheel vehicles (motorcycles, mopeds and bicycles), and vehicles for whom type of vehicle was not recorded were removed from the dataset in order to avoid confounding throughout the analyses. A sub-analysis comparing fatalities in these aforementioned vehicles to cars/trucks found that there was a 54% increased chance of a fatal outcome when a motorcycle, moped or bicycle were involved in a collision when compared to larger vehicles (OR = 1.54, 95% CI 1.07-2.20). Previous research suggests that this may be due to a decreased amount of protection when driving a two wheeled vehicle in addition to greater risk taking behavior profile shown by motorcycle drivers [13,14].

Previous literature has found that the time of the day of a collision is related to one’s chances of a fatal outcome [5,11]. In order to investigate this relationship, we chose to examine four-hour time periods in order to gain insight into specific time frames when driving patterns might be different, rather than examining broad categories of daytime, evening, and night.

Data included driver characteristics (e.g. age and sex), vehicle information (e.g. number of passengers and vehicle type), environmental (e.g. weather and road surface conditions) and highway characteristics (e.g. road characteristics, location on a highway, and highway number), as well as collision information (e.g. time and day) and severity of the collision which ranged from 0–4, but was dichotomized into death vs. no death for the analysis. Age groups were defined according to Erikson’s developmental stages [15]. Adolescent drivers were between 16 and 18 years old, early adults between 19 and 35 years old, middle adults between 36 and 65 years of age, and older adult drivers were defined as being older than 65 years of age. Road designs included: undivided 1-way, undivided 2-way (uses solid lines to keep directions separate), dividing barrier, divided (usually by an area of unpaved land), ramp road (part of an interchange allowing traffic to enter or exit the highway), collector (segment of highway which allows for entry and exit at interchanges), expressway, and transfer. We decided to include weather conditions in our analysis, even though it had not been previously identified as a risk factor, because Canadian weather conditions can be more extreme than those in some other parts of the world, and we wanted to examine if weather was associated with fatalities in Ontario.

After eliminating two-wheel vehicle cases (n = 1,395), a total of 52,131 vehicles involved in MVCs between 2001 and 2006 on the 400-series highways in Ontario were available for analyses.

### Statistical analyses

Descriptive statistics were calculated for nine variables: Highway name, Season, Day of the week, Time period, Sex, Age group, Environment/Weather, Road Characteristic, and Road Surface Condition; they were categorized into three groups: Driver Characteristics (Sex and Age group), Highway Characteristics (Highway name and Road Characteristic), and Environmental Characteristics (Season, Day of the week, Time period, Environment/Weather, and Road Surface Condition).

Chi-Square tests were conducted in order to detect differences in fatal injury rates among variables. Crude odds ratios (OR) and 95% confidence intervals (95% CI) were calculated using simple logistic regression in order to examine the relationships between each of the nine variables and a fatality as a result of the collision.

Finally, a multivariate analysis was conducted to estimate the odds of a fatality in each collision for the risk factors, adjusting for all other variables in the model, and for the clustering of vehicles in collisions where more than one vehicle was involved. This was done using the GENMOD procedure in SAS, with the unique identifier for each collision as the repeated variable. Initially, all nine risk factors were included in the model; however, collinearity between the Environment/Weather and Road surface condition variables was evident, therefore, we decided to eliminate the latter from the model. The outcome variable was if anyone (passenger or driver) died in the collision, compared to collisions where no-one died. Thus the numerator was the number of vehicles involving a fatality, while the denominator was all vehicles involved in collisions. Analyses were conducted using SAS v 9.0 [16].

## Results

Across all Ontario highways, the fatality rate among vehicles was 1.5%. This included 753 deaths among the reported 52,131 total vehicles involved in collisions. The distribution of fatality varied by age, sex, highway and environmental risk factors. Table 1 shows the distribution of non-fatal and fatal injuries among all categories for the eight included variables and Table 2 displays the results of the multivariate regression analysis.

**Table 1:** Distribution of fatal and non-fatal injuries by risk factor for 52,131 vehicles involved in collisions between 2001 and 2006 on Ontario’s 400-series highways

		**Outcome – N (%)**		**Total**
		**Fatal**	**Injury**	
Total		783 (1.5)	51,348 (98.5)	52,131
**Highway**				
	400	102 (1.9)	5,347 (98.1)	5,449
	401	456 (1.4)	31,094 (98.6)	31,550
	402	20 (3.4)	573(96.6)	593
	403	40 (1.3)	3,124 (98.7)	3,164
	404	13 (0.4)	3,088 (99.6)	3,101
	405	9 (15.8)	48 (84.2)	57
	406	18 (2,7)	640 (97.3)	658
	409	1 (0.3)	352 (99.7)	353
	410	10 (1.1)	907 (98.9)	917
	416	5 (1.2)	416 (98.8)	421
	417	82 (2.6)	3,013(97.4)	3,095
	420	3 (1.7)	170 (98.3)	173
	427	24 (0.9)	2,576 (99.1)	2,600
**Season**				
	Winter	218 (1.6)	13,762 (98.4)	13,980
	Spring	165 (1.4)	11,280 (98.6)	11,445
	Summer	238 (1.8)	12,890 (98.2)	13,128
	Fall	162 (1.5)	13,416 (98.5)	13,578
**Day of the Week**				
	Sunday	109 (1.7)	6,300 (98.3)	6,409
	Monday	114 (1.5)	7,292 (98.5)	7,406
	Tuesday	81 (1.1)	7,452 (98.9)	7,533
	Wednesday	98 (1.3)	7,290 (98.7)	7,388
	Thursday	103 (1.3)	7,624 (98.7)	7,727
	Friday	166 (1.9)	8,599 (98.1)	8,765
	Saturday	112 (1.6)	6,791 (98.4)	6,903
Time Period*				
	Evening (8 pm-12 am)	114 (2.0)	5,722 (98.0)	5,836
	Night (12 am-4 am)	146 (3.4)	4,137 (96.6)	4,283
	Early Morning (4 am-8 am)	99 (1.3)	7,619 (98.7)	7,718
	Morning (8 am-12 pm)	107 (1.1)	9,525 (98.1)	9,632
	Afternoon (12 pm-4 pm)	144 (1.4)	9,901 (98.6)	10,045
	Early Evening (4 pm-8 pm)	173 (1.2)	14,256 (98.8)	14,429
**Sex**				
	Female	176 (1.1)	15,464 (98.9)	15,640
	Male	607 (1.7)	35,884 (98.3)	36,491
**Age Group**				
	Adolescence	26 (1.1)	2,267 (98.9)	2,293
	Early Adulthood	363 (1.4)	25,742 (98.6)	26,105
	Middle Adulthood	326 (1.6)	19,483 (98.4)	19,809
	Late Adulthood	49 (2.3)	2,055 (97.7)	2,104
**Environment**				
	Clear	549 (1.4)	39,598 (98.6)	40,147
	Rain	45 (1.0)	4,567 (99.0)	4,612
	Snow	82 (1.5)	5,354 (98.5)	5,436
	Frozen Rain	23 (2.7)	842 (97.3)	865
	Drifting Snow	55 (12.8)	375 (87.2)	430
	Wind	10 (7.9)	117 (92.1)	127
	Fog	16 (4.1)	372 (95.9)	388
	Other	3 (2.4)	123 (97.6)	126
**Road Characteristic**				
	Undivided 1-Way	10 (1.3)	732 (98.7)	742
	Undivided 2-Way	42 (3.9)	1,026 (96.1)	1,068
	Dividing Barrier	318 (1.5)	21,463 (98.5)	21,781
	Divided	301 (5.0)	5,706 (95.0)	6,008
	Ramp Road	37 (0.6)	6,096 (99.4)	6,133
	Collector	30 (0.3)	8,799 (99.7)	8,828
	Expressway	43 (0.6)	7,067 (99.4)	7,110
	Transfer	2 (0.5)	416 (99.5)	418

*Missing data for: time period = 188, sex = 7, road characteristic = 43.

**Table 2:** Results of multivariate analyses for the odds of fatality among 52,131 vehicles involved in collisions between 2001 and 2006 on Ontario’s 400-series highways

**Risk factor**		AOR*	**95%CI**
**Highway**			
	401	1.00	ref
	400	0.97	0.67 - 1.39
	402	0.44	0.20 - 0.99
	403	0.48	0.29 - 0.80
	404	0.20	0.10 - 0.41
	405	6.89	2.16 - 21.98
	406	0.78	0.36 - 1.67
	409	0.20	0.03 - 1.49
	410	0.33	0.14 - 0.78
	416	0.25	0.08 - 0.70
	417	0.83	0.44 - 1.51
	420	0.68	0.09 - 5.21
	427	0.75	0.36 - 1.65
**Season**			
	Winter	1.00	ref
	Spring	1.23	0.84 - 1.82
	Summer	1.58	1.08 - 2.29
	Fall	1.62	0.71 - 1.54
**Day of the Week**			
	Sunday	1.00	ref
	Monday	1.06	0.66 - 1.70
	Tuesday	0.77	0.46 - 1.25
	Wednesday	0.97	0.61 - 1.55
	Thursday	1.00	0.64 - 1.57
	Friday	1.28	0.79 - 2.10
	Saturday	1.01	0.64 - 1.57
**Time Period**			
	Evening (8 pm-12 am)	1.00	ref
	Night (12 am-4 am)	1.67	1.07 - 2.59
	Early Morning (4 am-8 am)	0.63	0.39 - 1.00
	Morning (8 am-12 pm)	0.55	0.36 - 0.85
	Afternoon (12 pm-4 pm)	0.63	0.38 - 1.04
	Early Evening (4 pm-8 pm)	0.69	0.45 - 1.05
**Sex**			
	Male	1.00	ref
	Female	0.84	0.76 - 0.91
**Age Group**			
	Adolescence	1.00	ref
	Early Adulthood	1.52	1.01 - 2.29
	Middle Adulthood	1.82	1.20 - 2.75
	Late Adulthood	2.36	1.46 - 4.06
**Environment**			
	Clear	1.00	ref
	Rain	0.76	0.49 - 1.15
	Snow	0.93	0.57 - 1.51
	Frozen Rain	1.49	0.66 - 3.39
	Drifting Snow	7.39	2.21 - 24.78
	Wind	5.00	1.34 - 18.73
	Fog	2.78	1.11 - 6.96
	Other	1.72	0.34 - 8.58
**Road Characteristic**			
	Undivided 1-Way	1.00	ref
	Undivided 2-Way	3.74	1.37 - 10.28
	Dividing Barrier	1.28	0.54 - 3.03
	Divided	4.76	1.92 - 11.59
	Ramp Road	0.52	0.20 - 1.36
	Collector	0.27	0.09 - 0.79
	Expressway	0.44	0.17 - 1.26
	Transfer	0.37	0.04 - 3.13

* *AOR* = Adjusted Odds Ratio, *CI* = Confidence interval.

The analysis compared fatality rates in the four different age groups, broadly based on Erikson’s developmental stages [15]. An increasing trend was observed; as one got older, the fatality rate when involved in a MVC became greater. As shown in Table 1, the fatality rates were 1.1%, 1.4%, 1.6%, and 2.3% for teenagers, early adults, middle adults, and older adults respectively.

Multivariate analysis suggested that as age progresses, the odds of dying in a motor vehicle collision increases. Compared to teenaged drivers, cars with young adults drivers were more likely to suffer a fatality in a collision (AOR = 1.52; 95% CI, 1.01-2.29), middle and older adults drivers had an increased odds of being involved in a fatal crash (AOR = 1.82; 95% CI, 1.20 – 2.75) and 1.37% (AOR = 2.36; 95% CI, 1.46 – 4.06).

Sex was also associated with the odds of dying in a motor vehicle collision. Initial results showed that male drivers were involved in a collision with a fatal outcome 1.7% compared to 1.1% of cases for females. After adjustment for the other variables in the model, the odds of being involved in a fatal crash for cars driven by a female compared to those driven by a male were significantly lower (AOR = 0.84, 95% CI, 0.76-0.91).

### Highway characteristics

After adjustment, our analysis found that Highway 405 posed a significantly greater odds of fatality a 7-fold increased risk of fatal crash compared to Highway 401 (reference group) (AOR = 6.89, 95% CI 2.16-21.98). Seven of the 11 remaining highways showed a protective effect while the other 4 were not statistically different from Highway 401 (Table 2).

Undivided 2-way segments and divided 2-way segments without barrier showed higher fatality rates than segments with any other design (3.9% and 5.0% respectively). In multivariate analysis, the odds of fatality on an undivided 2-way design was three times greater than the risk on an undivided 1-way segment (OR =3.74 (95% CI, 1.37-10.28)). A divided road without a barrier was also associated with an increased odds of fatality(AOR = 4.76 (95% CI, 1.92-11.59)) compared to an undivided unidirectional design.

## Environmental characteristics

The distribution of collisions among all four seasons of the year (winter, spring, summer, and fall) was similar. However, fatalities seem to make up a higher percentage of collisions during the summer (30.4%). Initially, fewer collisions were reported in the summer months (n = 13,128) compared to the winter season (n = 13,980). However, once all other variables were added to the model, driving in the summer was associated with an increased odds of fatality compared to the wintertime (AOR = 1.58; 95% CI, 1.08-2.29).

Another purpose of the data analysis was to identify if there were any days of the week that had higher rates of fatality than others. The number of collisions per day of the week were similarly distributed (mean = 7,335). However, there appeared to be an increased risk of collision on Fridays, with a total of 8,566 over the span of six years. After adjusting for all other variables, no days showed significant differences in the odds of fatality, with Friday being associated with a slightly, but not significantly increased risk of death for those involved in MVCs (AOR = 1.28, 95% CI, 0.79 – 2.10).

From 4 am until 8 pm (early morning, morning, afternoon, and early evening), fatality rates amongst people involved in MVCs ranged from 1.2% to 1.4%. However, from 8 pm to 12 am (evening) it was elevated to 2.0% and during the nighttime (12 am to 4 am), it increased to 3.4%. Driving at night was associated with the highest risk of fatality compared to evening (AOR = 1.67, 95% CI, 1.07-2.59).

Finally, the association between weather condition and fatal MVCs on the 400-series highways in Ontario between 2001 and 2006 was examined. Fatality rates ranged from 1.4% in the ‘clear’ reference condition, to 12.8% under ‘drifting snow’ conditions. In the multivariate analysis, ‘drifting snow’, ‘wind’, and ‘fog’ were the only categories that remained significantly different from the ‘clear’ weather condition. ‘Drifting snow’ conditions were associated with an increased odds of death if involved in a MVC compared to MVCs in clear conditions (AOR = 7.39, 95% CI, 2.21-24.78). Windy conditions were associated with a greater odds (AOR = 5.00, 95% CI, 1.34-18.73) while fog was associated with an almost 3 times greater odds of fatality than clear conditions (AOR = 2.78, 95% CI, 1.11-6.96).

## Discussion

We found an increased risk of fatality as a result of a motor vehicle collision for older drivers and males. Further, undivided 2-way segments as well as divided 2-way segments were associated with increased fatality risk compared to divided 1-way segments. Driving on Ontario’s Highway 405 was associated with the highest odds of fatality in a collision. In addition, various environmental conditions were associated with an increased risk of dying as a result of a motor vehicle collision and they included driving in the summertime, at nighttime, and in drifting snow weather.

The results in this study found that an increase in driver age was strongly associated with an increased odds of fatality in the vehicle as a result of a motor vehicle collision. These findings are consistent with previous literature [5,10,11,17]. The magnitude of the effect age has on fatal outcomes varies throughout the literature; Bedard reported a five times higher risk of death among vehicles driven by 80+ year old drivers compared to middle aged counterparts and Singleton reported a 65% increase in chances of fatality when comparing adults drivers 60 years or older to younger 20 year old drivers [10,11]. Most importantly, the direction of the relationship remains constant; however, the magnitude may differ due to the use of different reference and comparison groups, different settings (city vs. highway MVCs), differing health practices in different geographical regions, and different adjustment for confounding variables in the multivariate analyses. The increased risk of fatality may be related to other age-related conditions, including co-morbid health conditions (e.g. epilepsy, dementia, diabetes mellitus, heart disease and hypertension) [5]. Observing the increased risk that older drivers are at, the Canadian Association of Occupational Therapists released the “National Blueprint for Injury Prevention in Older Drivers” in 2010, which by various methods, is designed to increase awareness of the issue. For example, the blueprint outlines various partnerships established to promote older driver safety throughout the country in addition to interventions that include media coverage, brochures for families with older adult drivers and information for health care practitioners taking care of them [18].

Our study suggested that female drivers were involved in fewer fatal motor vehicle collisions than men, a finding consistent with previous literature in which female drivers were 62% less likely to die in a collision compared to male drivers [19]. Further, Turner and McClure revealed that men scored significantly higher than females in the ‘Driver Aggression’ and ‘Risk Acceptance’ scales [19]. Lemieux reported an increased rate of fatalities amongst male drivers compared to females [12]. However, this finding is not completely consistent. For example, Bedard and Levine show up to a 50% increased risk for fatal injuries in women compared with men [10,20]. However, they state that this difference seems to be present only in younger drivers and that it disappears once older adults are added to the model.

Our results suggest that the undivided two-way design poses an increased fatality risk, as does the divided road with no barrier design; these results are consistent with previous research by Zhang [9]. Re-evaluating these two designs on Ontario’s highways, and installing barriers in both of these designs may improve road safety in some areas.

Further, our results showed that Highway 405 posed a significantly increased fatality risk when involved in MVCs on this 8.5 kilometer segment which connects the Queen Elizabeth Way to the Queenston-Lewiston Bridge (Canada-US Border). No previous research has been done examining specific highways and the risk involved in driving on them. Although we have no evidence for why the fatality rate is higher than on most other highways, we hypothesize that there may be an elevated number of trucks on this highway because it is one of the arteries that connects Canada to the United States and therefore heavy transport vehicles are common. Collisions involving large trucks have been shown to be deadly more often than cars and small trucks [21]. This possibility warrants further investigation.

Our finding that summer was associated with a significantly higher likelihood of fatality is consistent with Zhang who reported that young drivers were at a 25% increased risk for fatality compared to middle aged drivers involved in MVCs during the summer [5].

The lack of association between day of the week and fatal collisions on Ontario’s highways is not consistent with previous research by Lemieux who reported that MVCs in the Hamilton-Wentworth Niagara Region of Ontario that resulted in fatalities occurred during Fridays and Sundays, on city and rural roads as well as highways [12]. Other studies compared fatality rates in different days of the week; however, they compared weekdays vs. weekend days rather than each day individually [5,17,22].

Similarly to Valent, who found that deaths increased as a results of MVCs between 1:00 and 5:00 a.m. by a factor of two, this study found that night (0:00–4:00 am) was the most fatal period of the day with a 67% higher risk of fatal MVCs than the evening [17]. Possible reasons for an increased occurrence of fatal accidents during the nighttime/early morning hours include an increased use of alcohol and/or drugs while driving and tiredness or drowsiness while driving during these hours [23,24].

We found that ‘drifting snow’, ‘wind’, and ‘fog’ were significantly associated with an increased fatality risk. A study by Khattak et al. which compared collision rates on American Interstate freeways for periods with snowy conditions to those with clear weather found that during snowstorms the crash rate was 5.86 crashes per million vehicle kilometers (vs. 0.41 during clear conditions). These results represented a 13 times increased collision risk during snowy weather [25]. These findings complement those by Qui and Nixon (2008) although their systematic review found that in the last four decades, the effect of adverse weather on fatal collisions has decreased substantially [26].

## Strengths and limitations

The main strength of this study is that it was able to examine the relative contribution of driver-related and environmental risk factors to fatal motor vehicle collisions on Ontario’s 400-Series Highways. All major provincial highways were included in the analyses and the most comprehensive source for MVC data was used (Ministry of Transportation of Ontario) whose database included 96.1% of cases with no missing data, leaving only 3.9% of cases ineligible for some of the analyses.

Vehicles which were evidently at an increased risk for fatality when involved in a MVC (e.g. motorcycles and bicycles) were excluded in order to allow the analyses to approximate a more accurate fatality risk posed to occupants of vehicles with similar safety mechanisms and driving conditions.

The limitations of this study include a potential for misclassification bias since the data is entirely based on police reports and non-fatal crashes may therefore be underreported. Also, the reports only include outcome data up to 30 days after the collision. Therefore, collisions that resulted in fatalities 30 days after they took place would have not been classified as fatal collisions. We had no information on driver distraction, kilometers driven, or other potentially confounding variables not included in the database, including use of restraints and impaired driving, and access to trauma care. However, we believe it is unlikely that these variables are systematically different across different highways. The results of this study may only be generalizable to other jurisdictions with similar highway structures. Finally, the most recent data available were from 2006 and there have been improvements to the highways since then. These improvements include widening Highway 401 and 417, and improvements to bridges. Future research can evaluate the impact of these changes.

## Conclusions

Even after controlling for characteristics of the driver, the highway and the environment contribute to the likelihood of someone dying in a motor vehicle collision. Interventions to reduce deaths may focus on structural road redesign, as well as driver-related interventions targeted at reviewing driving practices within different age groups and for drivers under different conditions. Our research suggests that both driver-level and environmental interventions may help reduce the risk of fatality in a motor vehicle collision.

## Competing interests

The authors declare that they have no competing interests.

## Authors’ contributions

DR participated in study design, obtained study data, performed the statistical analysis, drafted, and edited the manuscript. HT participated in study design, reviewed statistical analysis, aided in draft and final manuscript compilation. AM conceived of the study, designed the study, performed the statistical analysis, drafted and edited the manuscript. All authors read and approved the final manuscript.

## Pre-publication history

The pre-publication history for this paper can be accessed here:

http://www.biomedcentral.com/1471-2458/12/1125/prepub
